# Baseline Association between Healthy Eating Index-2015 and Health-Related Quality of Life in Breast Cancer Patients Enrolled in a Randomized Trial

**DOI:** 10.3390/cancers16142576

**Published:** 2024-07-18

**Authors:** Giuseppe Porciello, Sergio Coluccia, Sara Vitale, Elvira Palumbo, Assunta Luongo, Maria Grimaldi, Rosa Pica, Melania Prete, Ilaria Calabrese, Serena Cubisino, Concetta Montagnese, Luca Falzone, Valentina Martinuzzo, Luigina Poletto, Emanuela Rotondo, Piergiacomo Di Gennaro, Michelino De Laurentiis, Massimiliano D’Aiuto, Massimo Rinaldo, Guglielmo Thomas, Francesco Messina, Francesca Catalano, Francesco Ferraù, Vincenzo Montesarchio, Diego Serraino, Anna Crispo, Massimo Libra, Egidio Celentano, Livia S. A. Augustin, the DEDiCa Study Group

**Affiliations:** 1 Epidemiology and Biostatistics Unit, Istituto Nazionale Tumori IRCCS “Fondazione G. Pascale”, 80131 Naples, Italy; sergio.coluccia@istitutotumori.na.it (S.C.); sara.vitale@istitutotumori.na.it (S.V.); elvira.palumbo@istitutotumori.na.it (E.P.); assunta.luongo@istitutotumori.na.it (A.L.); m.grimaldi@istitutotumori.na.it (M.G.); r.pica@istitutotumori.na.it (R.P.); melania.prete@istitutotumori.na.it (M.P.); e.rotondo@istitutotumori.na.it (E.R.); e.celentano@istitutotumori.na.it (E.C.); l.augustin@istitutotumori.na.it (L.S.A.A.); 2 Healthcare Direction, “A. Cardarelli” Hospital, 80131 Naples, Italy; ilariacalabrese@live.it; 3 Humanitas Istituto Clinico Catanese, 95045 Misterbianco, Italy; serena-cubisino@hotmail.it; 4 Institute of Food Science, CNR Italy, 83100 Avellino, Italy; concetta.montagnese@isa.cnr.it; 5 Department of Biomedical and Biotechnological Sciences, Oncologic, Clinical and General Pathology Section, University of Catania, 95124 Catania, Italy; luca.falzone@unict.it; 6 Cancer Epidemiology Unit, National Cancer Institute, CRO, IRCCS, 33081 Aviano, Italy; nutrizionista.martinuzzo@gmail.com (V.M.); poletto.gina08@gmail.com (L.P.); serrainod@cro.it (D.S.); 7 Medical Statistics Unit, University of Campania “Luigi Vanvitelli”, 80138 Naples, Italy; piergiacomo.digennaro@unicampania.it; 8 Department of Breast and Thoracic Oncology, Division of Breast Medical Oncology, Istituto Nazionale Tumori IRCCS “Fondazione G. Pascale”, 80131 Naples, Italy; m.delaurentiis@istitutotumori.na.it; 9 Clinica Villa Fiorita, 81031 Aversa, Italy; massimiliano.daiuto@gmail.com (M.D.); massimo.rinaldo@tiscali.it (M.R.); 10 Clinica Mediterranea, 80122 Naples, Italy; guglielmo.thomas@outlook.it; 11 Ospedale Evangelico Betania, 80147 Naples, Italy; messina52@alice.it; 12 Cannizzaro Hospital, 95126 Catania, Italy; fcatalano1968@tiscali.it; 13 Ospedale San Vincenzo, 98039 Taormina, Italy; ferrau@oncologiataormina.it; 14 UOC Oncologia, AORN dei Colli (Monaldi-Cotugno-CTO), 80131 Naples, Italy; vincenzo.montesarchio@ospedalideicolli.it (V.M.); m.libra@unict.it (M.L.)

**Keywords:** healthy eating index, diet quality, health-related quality of life, breast cancer survivors

## Abstract

**Simple Summary:**

Quality of life significantly affects health outcomes in cancer patients. However, evidence of an association between diet quality and quality of life in cancer survivors is sparse in Mediterranean countries. The aim of this study was to evaluate the associations between an a priori diet quality index, the Healthy Eating Index-2015 (HEI-2015), and quality of life, assessed through a validated questionnaire targeted at women with a breast cancer diagnosis. A higher HEI-2015 score was positively associated with summary quality of life score and inversely associated with symptom scores.

**Abstract:**

Health-related quality of life (HRQoL) represents one of the most concerning aspects for cancer patients. The Healthy Eating Index (HEI) is an a priori diet quality index directly associated with health outcomes and HRQoL in cancer survivors in North American populations. We evaluated, in a Mediterranean population, the baseline associations between HEI-2015 and HRQoL in 492 women with breast cancer recruited in a DEDiCa lifestyle trial. Dietary data were obtained from 7-day food records; HRQoL was assessed through the European Organisation for Research and Treatment of Cancer Quality of Life Questionnaire Core 30 (EORTC QLQ C30) and the C30 Summary Score (SumSc). Analysis of variance and multivariable linear and log-gamma regression models were performed. Mean and standard deviation for HEI-2015 score was 68.8 ± 11.2; SumSc was 81.5 ± 12.9. Women with lower HEI-2015 score had higher BMI, were more frequently exposed to tobacco smoke and had fewer years of education. Patients with a HEI-2015 score greater than 68.7 (median value) showed a significant increase in SumSc of 4% (*p* = 0.02). HEI-2015 components also associated with SumSc were beans and greens (β = 1.04; *p* = 0.02). Weak associations were found for total vegetables and saturated fats. Higher diet quality in breast cancer survivors was associated with higher overall HRQoL in this cross-sectional analysis.

## 1. Introduction

The Global Cancer Observatory’s latest updated data (GLOBOCAN 2022 [1]) indicate that worldwide 19.9 million new cases of cancer have been estimated, representing the chronic disease with the highest psychological and socio-economic impact. People who get cancer undergo significant physiological change, emotional stress and economic difficulties [1,2]. Most cancer patients report treatment side effects which impact their overall health, including mental and physical disorders, which contribute to poor compliance and prognosis [3,4].

Breast cancer (BC) is the most prevalent female cancer globally, with 2.3 million cases in 2022 (23.8% of total), followed by lung cancer (about 900 thousand new cases, 9.4%), with 57.000 new cases estimated in Italy [1,5]. The onset of BC is not only motivated by unmodifiable factors, such as age, genetics and family history, but also by modifiable factors that are affected by a patient’s lifestyle, such as reproductive history, hormone therapy, alcohol consumption, dietary habits and physical activity [6,7]. A BC diagnosis may result in physical and mental distress and substantial changes in daily life and family dynamics [8]. Evidence indicates that BC survivors may experience several physical and mental disorders, including fatigue, pain, and anxiety [9,10,11,12,13,14]. The quality of life and life expectancy of BC patients can be negatively affected by the side effects of oncologic treatments on cognitive function, specifically anxiety, depression, fear of recurrence, psycho-physical stress, lack of concentration, memory loss, disease-related cognitive fog (“chemobrain”) and sleep disturbances [15,16,17].

According to the World Health Organization (WHO), health-related quality of life (HRQoL) is an important self-perceived parameter of patients’ general health, providing information on physical, psychological and emotional characteristics and social appearance [18,19]. HRQoL assessment in cancer patients provides important information to clinicians, representing a primary endpoint in health and medical research. Cancer-related factors and patient’s lifestyle habits, such as smoking, sedentary lifestyles and poor-quality diet can influence HRQoL [20,21,22,23]. In breast cancer patients, the presence of comorbidities such as hypertension, diabetes and osteoarthritis has been associated with poor quality of life, especially physical dysfunction and body pain [24].

Healthy dietary patterns including the Mediterranean diet (MedDiet) have been associated with HRQoL. Data from BC survivors living in Italy show positive associations between higher adherence to the Mediterranean Diet (MedDiet) and HRQoL both in the cross-sectional baseline analysis and at one year after lifestyle intervention [22,23]. In a Chinese cohort of women with early-stage BC, a higher consumption of a vegetable and fruit dietary pattern improved quality of life [25,26]. The role of diet quality on chronic disease progression is becoming increasingly important also for cancer. Poor nutritional intakes are common in cancer patients as a consequence of cancer-related symptoms and anti-cancer treatments [27]; therefore, diet quality assessment for cancer survivors is a health priority [26,28]. To assess the quality of the overall diet, different dietary quality indices (DQIs) have been developed over the years: they consist of a priori and a posteriori quality indices [29]. A priori indices are based on algorithms associated with nutritional recommendations; a final score is associated with higher or lower adherence to a predefined dietary pattern or known dietary guidelines. A posteriori indices are derived from statistical and technical models such as cluster and factor analysis. A priori indices are faster to calculate and can be used to study the effects of diet on pathologies [30]. Food quality indices or indicators (DQIs) are algorithms aimed at evaluating how the overall diet correlates with dietary guidelines. The Healthy Eating Index-2015 (HEI-2015) was designed to align with the 2010–2015 Dietary Guidelines for Americans (DGA) which recommended the consumption of vegetables, fruit, cereals, legumes including soybeans and lean meats, as well as a reduced intake of saturated fats, sugar, sodium and alcohol [31]. Although the evidence supports a direct association between diet quality and quality of life in healthy subjects and patients with different diseases, these studies showed high heterogeneity in HRQoL measurements, definitions of a healthy dietary pattern (a dietary pattern rich in fruits and vegetables, MedDiet), dietary assessment methods and insufficient adjustments for confounders. Moreover, in these studies, different DQIs were used, calculated a priori or a posteriori [22,25,32,33,34,35,36,37].

Furthermore, most of the evidence comes from studies conducted in non-Mediterranean countries characterized by dietary patterns that do not fully adhere to a Mediterranean dietary pattern. Finally, to date, we have not found studies that investigated the association of HRQoL evaluated as a unique index (Summary Score, SumSc) calculated from validated questionnaires and DQIs in BC survivors. Therefore, our aim was to evaluate a possible association between a priori diet quality index (HEI-2015) and quality of life, as individual scales and as a unique overall index, in BC patients living in the Mediterranean region.

## 2. Materials and Methods

### 2.1. Study Design

This investigation is part of an ongoing Italian multicenter randomized controlled study, started in 2016 (DEDiCa Study) [38]. The protocol was approved by the Italian Ministry of Health, Italian Medicines Agency—AIFA (EudraCT Number 2015-005147-14) and by the Ethics Committee of each recruiting center (ClinicalTrials.gov identifier NCT02786875). It investigates the effect of a low glycemic index diet, physical activity and vitamin D supplementation on breast cancer recurrence [38]. Four hundred and ninety-two patients were recruited, between November 2016 and July 2021, in the involved cancer research institutes and departments located in Italy, specifically in the Istituto Nazionale Tumori IRCCS Fondazione G. Pascale (Naples), Clinica Mediterranea (Naples), Villa Betania (Naples), Cannizzaro Hospital (Catania), San Vincenzo Hospital (Taormina), Istituto Nazionale Tumori IRCCS CRO (Aviano), and the AORN dei Colli (Monaldi-Cotugno-CTO) of Naples. Inclusion criteria were the following: women with a primary diagnosis of non-metastatic breast cancer confirmed by histology, Ki-67 ≥ 30%, aged between 30 and 74 years, able to understand and sign informed consent and to adhere to a study program, including visits and treatment. All patients who did not meet these criteria were excluded; in addition, patients diagnosed with other cancers, severe renal failure, hypercalcemia, kidney stones, granulomatous diseases or sarcoidosis were excluded. A blood sample for routine analysis was collected at the baseline for each participant. Patient’s anamnestic, dietary, quality of life and anthropometric data were collected at baseline visit and are described in the paragraphs below. Weight and height of study participants were obtained by trained staff at the baseline study visits. Height was measured to the nearest 1 cm using a Seca stadiometer, and weight was measured to the nearest 0.5 Kg using a Seca scale (Seca 761). Body mass index (BMI) was calculated using the formula weight (kg)/height (m^2^). We collected information on weekly physical activity through an electronic pedometer (Omron Walking Style IV) worn by patients for one week prior to each study visit, and through an anamnestic questionnaire produced by study staff investigating physical activity at home, at work and during free time.

### 2.2. Dietary Intake

Food intake was collected through 7-day food diaries; these were completed one week before baseline visits. We provided standard templates and guides to help patients fill in their food diaries as accurately as possible. WinFood© Entry 3.11.0, Medimatica software was used to insert information from food diaries and extract daily mean intakes of macronutrients, micronutrients, dietary fiber, calories and food groups in grams per day. This nutritional software is based on the database provided by Istituto Nazionale di Ricerca per gli Alimenti e la Nutrizione (CreaNUT) [39] and the Food Composition Database for Epidemiological Studies in Italy (Banca Dati di Composizione degli Alimenti per Studi Epidemiologici in Italia—BDA) [40].

### 2.3. Healthy Eating Index-2015 (HEI-2015)

The HEI-2015 is a valid tool to assess diet quality, evaluating how well a set of foods consumed aligns with Dietary Guidelines for Americans (DGA) [31]. These recommendations promote consumption of vegetables, fresh fruits, beans, nuts, whole grains, low-fat dairy products and unsaturated vegetable oils while limiting saturated fatty acids and added sugars [41]. The HEI-2015 is made of 13 components, 9 representing healthy foods. For each HEI component, intakes of foods and nutrients are represented on a density basis, counted as amount per 1000 kcal. From weekly food diaries obtained from patients, specific foods and food groups in grams were calculated. Grams were converted into equivalent cups and ounces before specific HEI-2015 components were calculated, using the Food Patterns Equivalents Database (FPED). FPED is a research tool that evaluates food and drink intakes against the DGA recommendations [42]. The HEI-2015 total score is the sum of the adequacy components and moderation components and ranges from 0 (low adherence to the DGA) to 100 (highest adherence to DGA). Adequacy components (total fruit, whole fruit, total vegetables, greens and beans, whole grains, dairy, total proteins, seafood and plant proteins and fatty acids) represent food groups or dietary elements that are encouraged. Higher scores indicate higher assumptions, which are desirable. Moderation components include refined grains, sodium, added sugars and saturated fats: for these, limited consumption is recommended, and higher scores represent lower consumption, and are therefore desirable. Supplementary Table S1 and Figure S1 provide information on the construction of the model and characteristics of the food groups in the HEI-2015. All specific features, including the calculation algorithm, have been published and updated on Epidemiology and Genomics Research Program—National Cancer Institute website [43].

### 2.4. Health Related Quality of Life: EORTC QLQ-C30 and C30 Summary Score

Quality of life was assessed at baseline with the validated European Organisation for Research and Treatment of Cancer Quality of Life Questionnaire Core 30 (EORTC QLQ-C30). This questionnaire measures functional aspects of quality of life and symptoms commonly reported by cancer patients. EORTC QLQ-C30 includes five functional scales (physical, role, cognitive, social and emotional), nine symptom scales (fatigue, nausea or vomiting, pain), with six individual items (dyspnea, sleep disturbance, appetite loss, constipation, diarrhea and financial impact) and global health status/quality of life item, formed by two questions concerning patient’s overall quality of life. The questionnaire consists of 30 questions, and each cancer patient can answer these questions regardless of therapy performed or cancer type. Twenty-eight questions have four possible answers: 1 = Not at all, 2 = A little, 3 = Quite a bit, 4 = Very much. Two questions have a Likert scale from 1 to 7 as answers. All scores were linearly transformed into a 0–100 scale. Scores were calculated using the EORTC QLQ-C30 Scoring Manual 3rd Edition [44,45]. For functional scales, higher scores indicate better health status; for symptoms, higher scores indicate worse health status. To use a unique index representing global quality of life, the C30 Summary Score (SumSc) ranging from 0 to 100, was obtained as the mean values of functional and symptomatic scales from the EORTC QLQ-C30 questionnaire according to a validated methodology [46]. To calculate the mean score, symptoms scales were reversed to obtain uniform scales; therefore, we subtracted 100 from the symptom scales. The non-missing scores of transformed scales were then added to the non-missing functional scales’ scores and were finally divided by the count of non-missing scores (up to 13), excluding those from calculations regarding the global health status/QOL and financial difficulty. The detailed formula is as follows: (13 − k)^−1^ Σ (Physical Functioning, Role Functioning, Social Functioning, Emotional Functioning, Cognitive Functioning, (100-Fatigue), (100-Pain), (100-Nausea and Vomiting), (100-Dyspnoea), (100-Insomnia), (100-Appetite Loss), (100-Constipation), (100-Diarrhoea), where k is the count of missing answers [46,47].

### 2.5. Statistical Analysis

The participants’ general characteristics (age, smoking status, civil status, education, surgery type, cancer treatments at baseline, cardiometabolic risk factors, molecular subtypes, time since surgery, BMI, step count) were calculated as means, standard deviations, counts and percentages. Menopausal status was self-reported; geographic area was based on recruitment center; time since surgery was dichotomized as more or less than 8 months; type of surgery was either quadrantectomy or mastectomy; the categories of cancer therapies (chemotherapy neoadjuvant/adjuvant, radiotherapy, hormone therapy) were never, ongoing, or completed; the time since last chemotherapy was dichotomized as less than two months, more than two months. We used SAS code for the HEI-2015 [48], read by R software, to calculate HEI-2015 total score and HEI-2015 nutritional components. To assess HEI-2015, a specific algorithm was used [3]; mean, median and quartiles were calculated. To describe patients’ characteristics according to low and high values of HEI-2015 (by median values), a Wilcoxon rank test was used. From EORTC QLQ-C30 questionnaires results, mean values, standard deviation, median and interquartile ranges were calculated for each dimension, according to the manual. A multivariable linear regression model (LM) was performed to estimate the adjusted relationships between HEI-2015 and EORTC QLQ-C30 functional and symptom scales. A multivariable log-gamma regression model (LG) was performed to evaluate the association between the SumSc with HEI-2015 total score and its components. Median values (Me) were calculated for each of these covariates. β’s referred to over-median group risk for HEI and each other component, and they were shown as exponentials of LG coefficients, i.e., Exp(β). Finally, a multivariable LG regression model (adjusted for smoking status, physical activity (<2500, ≥2500 steps/day), time since surgery (<8, ≥8 months), cardiometabolic risk factors (none, 1, ≥2), cancer stage (I, II and III), chemotherapy status (ongoing, never, <2 months, ≥2 months), radiotherapy status (ongoing, never, finished), and menopausal status (menopausal, perimenopause, premenopausal)) was performed to assess mean adjusted risk factors between SumSc with HEI-2015 and cancer therapies. R software (v. 4.3.2), IBM SPSS Statistics version 25.0 and Microsoft Excel 2016 were used for the analyses.

## 3. Results

### 3.1. General and Demographic Baseline Characteristics

Mean age of participants was 52.3 ± 9.11 years, 80.4% were married, 20.5% were smokers and 63.5% had more than 10 years of school education; mean BMI was 27.7 ± 5.7 (61.4% with BMI > 25 kg/m^2^), 70% reported less than 5000 steps per day; 75.8% underwent quadrantectomy, and the remaining underwent mastectomy; 10.0% underwent and finished neoadjuvant chemotherapy, and 17.7% underwent adjuvant chemotherapy, 45.9% of which had finished it before baseline; 9% underwent radiotherapy, 51.4% of which had finished it before baseline; 86.4% had stage I-II BC, 13.6% had a stage III BC. BC molecular subtypes were identified with the following prevalence: 49.0% luminal B subtype, 32.7% luminal A, 14.2% triple negative and 4.1% HER2 positive.

### 3.2. HEI-2015 Scores

All indices have been calculated according to the specifications defined by the original method [31,41,42,43,48]. Mean HEI-2015 score for the 492 BC patients was 68.8 ± 11.2 out of 100, representing the highest diet quality or total adherence to guidelines, with a minimum value of 28.7 and a maximum of 98.2 (median 68.7). Higher scores for individual components (above 80% of the maximum score) were for greens and beans (4.2 ± 1.5 out of 5; 84%), total fruit (4.0 ± 1.5 out of 5; 80%), whole fruit (4.3 ± 1.4 out of 5; 86%), total proteins (4.6 ± 0.8 out of 5; 92%) and seafood and plant proteins (4.4 ± 1.2 out of 5; 88%) defined as adequacy components: for these, higher scores reflect higher intakes, which are desirable in this case. The highest scores for moderation components (dietary components to decrease) were for sodium (9.4 ± 1.5 out of 10; 94%) and added sugars (9.9 ± 0.2 out of 10; 99%): for moderation components, higher scores reflect lower intakes, which are more desirable. Mean scores for each HEI component (total vegetables, greens and beans, total fruit, whole fruit, whole grains, dairy, total protein, seafood and plant proteins, fatty acids, sodium, refined grains, added sugars, saturated fats) and mean percentages of the maximum score are shown in Table 1.

### 3.3. Characteristics of the Study Participants by HEI-2015 Median Values

Table 2 shows the distribution by low and high HEI-2015 scores of patients’ characteristics (including age, BMI, smoking status, civil status, step count, cardiometabolic risk factors, cancer stage, time since surgery, surgery type, neoadjuvant chemotherapy, adjuvant chemotherapy, radiotherapy). Women with lower HEI-2015 score (<68.7) had higher BMI, were more frequently exposed to tobacco smoke and had fewer years of education.

### 3.4. Health Related Quality of Life (HRQoL) and HEI-2015 Score

Table 3 shows the mean scores of the EORTC QLQ C30 dimensions. Among quality of life’s functional scales, physical and cognitive functioning were highest, at 82.6 ± 15.5 and 82.5 ± 20.3, respectively. On the other hand, among symptoms scales where higher values correspond to a worse health status, the highest scores were for fatigue (32.4 ± 22.6), pain (23.4 ± 22.1) and insomnia (29.3 ± 27.2). The SumSc, obtained from EORTC QLQ C30, was 81.5 ± 12.9. The median values and the interquartile ratios are shown in Supplementary Figure S2.

In the LM models, significant associations were observed between HEI-2015 and QoL scores: pain (*p* = 0.02), fatigue (*p* < 0.01), dyspnea (*p* < 0.01), diarrhea (*p* < 0.01) and financial difficulties (*p* = 0.01) for symptoms scales. A weak significant association was also found between physical and social functioning and HEI-2015 score (*p* = 0.08 and *p* = 0.05). Beta coefficients are shown in Table 3.

In the LG models, significant associations were observed between the HEI-2015 score and its components and the EORTC QLQ C30 SumSc: in particular, patients with a HEI-2015 score greater than 68.8 (median value), showed a 4% increase in SumSc (β = 1.04, 95%CI: [1.01, 1.07]; *p* = 0.02). Among HEI components, significant associations were found with beans and greens (β = 1.04, 95%CI: [1.01, 1.07]), for which increased consumption is associated with improved quality of life (SumSc increased by 4%). Weakly and borderline significant associations were also observed both for total vegetables (β = 1.02, 95%CI: [1.00, 1.05]; *p* = 0.08) and saturated fats (β = 0.97, 95%CI: [0.95, 1,00]; *p* = 0.06) (Table 4).

Table 5 shows a positive association between HEI-2015 score and SumSc, indicating that better food quality (HEI-2015 ≥68.7) increases quality of life by 3% (*p* < 0.05). Moreover, physically active patients experienced +5% and +9% increases in SumSc compared to those more sedentary (*p* < 0.001). Lastly, factors that could influence the association between HRQoL and diet were chemotherapy (CT) and radiotherapy (RT): compared to patients who had received CT treatment, patients who finished at least two months before baseline assessment showed a 9% higher quality of life SumSc (β = 1.09, 95%CI: [1.03, 1.15]); similarly, those who had not started chemotherapy showed a 6% higher score (β = 1.06, 95%CI: [1.02, 1.12]) than those who had started chemotherapy before the baseline assessment. Patients who had not received (β = 1.05) or who completed RT (β = 1.08), showed a better SumSc score compared to those who were receiving RT, but with a borderline significance (*p* = 0.05) (Table 5).

## 4. Discussion

In this study we aimed to evaluate the relationship between diet quality using an a priori DQI (HEI-2015) and HRQoL in women living in a Mediterranean country with non-metastatic breast cancer. We found significant inverse associations between HEI-2015 scores and the symptomatic scale, fatigue, pain, dyspnea, diarrhea and financial difficulties. A significant positive association was also confirmed in subsequent multivariate analyses between SumSc and diet quality.

Women who are diagnosed with breast cancer undergo severe emotional shock and are forced to adapt to major physical and emotional changes [49,50]. Breast cancer survivors suffer from multiple symptoms that hinder a good quality of life, especially fatigue and pain following adjuvant or neoadjuvant therapy and surgery, which can reduce the use of an affected arm and shoulder [51]. Quality of life assessment in breast cancer patients has become central in recent years [52], using tools that are able to validly quantify the functional and psychological aspects of patients [53]. SumSc that include all functional dimensions (emotional, physical, social, etc.) and symptoms (pain, fatigue, insomnia, etc.) seem to be more efficient and reliable in oncology research [46,47,54]. Adherence to healthier dietary patterns, and hence a high Healthy Eating Index score, has been inversely associated with the overall risk of mortality in cancer patients, particularly for post-diagnosis breast cancer [55,56]. The indices used to evaluate the diet quality can be used as cancer prevention tools, but also represent very useful tools to assess the possible association of diet with diet-related pathologies (diabetes, obesity, cancer), especially in different populations and societies in the world with different habits [57,58]. Higher diet quality has improved post-menopausal breast cancer survivors’ quality of life [59]. The impact of diet quality on post-diagnosis quality of life has been evaluated through various methods [33]; however, none included the summary score (SumSc) in association with diet quality, although SumSc may have higher prognostic value compared to single scales or global QoL [46].

Direct associations between diet quality and quality of life status were found in a Chinese cross-sectional study [25]. In this study, 1462 patients were enrolled within 12-months post-diagnosis; for the analyses, the complete data of 1226 patients was used. Data from an FFQ questionnaire were used to assess diet quality, while the EORTC QLQC30 questionnaire was used to evaluate quality of life. Generalized estimation equations were used to evaluate longitudinal associations between dietary patterns and QoL. Researchers have indicated an association between dietary patterns and some aspects of quality of life: a higher consumption of grain and animal-based foods was inversely associated with role functioning, dyspnea and constipation. Higher intake of vegetables was associated with better quality of life in terms of GHS score, physical and emotional functioning, and reduced sleep disturbances and diarrhea. In our study, similarly, we saw that a higher HEI score, including increased consumption of plant foods, being fruits and vegetables, was associated with reduction of symptoms such as diarrhea and improved quality of life.

A pilot cross-sectional study showed an association between mental quality of life (MHRQoL) and nutritional status, using HEI-2015 as DQI [60]. The study included 90 breast cancer patients with a mean age of 71 years, with a mean BMI of 28.2, similar to our sample. MHRQoL consists of a composite index of emotional wellbeing and social functioning, obtained from RAND-36 (63.6 ± 10.2 and 83.5 ± 19.6, respectively). These authors confirmed a significant positive association between HEI-2015 and social functioning and emotional wellbeing in breast cancer survivors [60]. Using EORTC QLQ C30, we found a weak direct association between diet quality (HEI-2015) and emotional and social functioning. Associations between mental health and nutritional aspects confirm once again the importance of specific and targeted counselling for cancer patients to reduce anxiety and depression.

The mean HEI-2015 score calculated for 492 BC patients in our study was 69.0. The National Health and Nutrition Examination Survey 2005–2016 (NHANES) for American adult cancer survivors reported an average HEI score of 58.9 in women with BC, indicating a low diet quality among American adult cancer survivors [61]. As indicated in several studies that used HEI-2015, scores were divided into three categories: score ≤ 50 was described as “poor diet quality”, scores of 51–80 were considered “needs improvement” and scores of >80 indicated “good diet quality”. In a cross-sectional study of 52 cancer survivors, the mean HEI-2015 score was 56.2 for BC survivors, with a range from 29.7 to 84.6 [62], which is lower than the mean for the American population [63]. In a cross-sectional study conducted in Malaysia, the mean HEI-2015 score in 179 BC patients was 63.9 [64]. In a case-control study of BC patients, using an earlier version of HEI (HEI-2005 [65]), the score was lower in pre-menopausal BC patients vs. matched controls (64.8 ± 9.7 vs. 67.9 ± 8.8, respectively; *p* < 0.001) [66]. Compared to other studies, the mean HEI-2015 score in our study tended to be higher, possibly because this study was carried out in Italy where consumption of the Mediterranean diet is widespread and so are the typical components of a healthy diet. In our sample, 41% of BC patients showed a low adherence to the Mediterranean diet at baseline.

In our study, there was a significant association between years of education and HEI-2015 score as previously found by others [67,68]. A longer period of education may be linked to a better knowledge of healthy foods and understanding of the value of nutritional aspects, especially in pathological conditions. Indeed, patients with higher levels of education are generally found to be more informed about the best choice of healthy foods and more interested in nutrition education [69,70,71]. So, our analysis confirms the importance of educational meetings and on-going updating with experienced staff aimed at improving nutritional knowledge [72] and possibly post-diagnosis overall health, including quality of life [23,73,74]. A significantly lower HEI-2015 score was also found in smoking patients compared to non-smokers; these results are in line with observational studies investigating diet quality in smokers [75]. HEI-2015 score has been found to be significantly higher in patients with normal weight (<25 kg/m^2^ BMI) compared to patients who are overweight or obese. Previous studies suggested this association between a high-quality diet and BMI [76], particularly one study which evaluated HEI-2015 and its relationship with BMI within a multiethnic cohort over 20 years (mean age 48 years), that indicated an inverse association between HEI-2015 and BMI across time.

Among other validated indices assessing overall diet quality, the HEI-2015 is one of the commonly a priori-defined indices. Other studies confirm the association between the HEI-2015 and a reduced risk of breast cancer, indicating the relevance of HEI-2015 as a measure of diet quality in cancer prevention [77]. Specifically, our results also confirm that a healthy diet, rich in vitamins, minerals and other components present in vegetables, fruits, whole grains and nuts, have antioxidant, anti-inflammatory and anti-proliferative effects, and therefore should be preferentially consumed by cancer patients [78]. In this work a higher dietary quality index was associated with a lower degree of pain and fatigue, showing once again that high consumption of plant-based foods (vegetables, fruits, whole grains, legumes, nuts, olive oil) and low or limited consumption of red meat, milk, and sweets were linked to fewer pain and fatigue symptoms in cancer survivors after diagnosis. The results of this study can help us to confirm that the use of validated indices give us a measure of the quality of the diet in breast cancer patients, but above all it helps us to understand how the dietary assessment of the patient and its improvement can impact their quality of life, prognosis, and survival. For clinical researchers, it is complicated to define which is the most important prognostic domain among those obtained from quality-of-life questionnaires, such as physical functioning, fatigue or pain. Therefore, a unique summary index such as SumSc could be better understood.

Despite the strengths and confirmations of this analysis, there are several limitations: being a cross-sectional analysis of baseline data from a medium size trial, it is not representative of the whole population of breast cancer survivors in Italy. However, being a randomized controlled trial, data were accurately collected and checked, including 7-day diet records. Validated QoL questionnaires were completed by patients who may have altered perceptions of symptoms or physical states, thus providing an answer that is not totally correct. There were several strengths to our study. Detailed 7-day food diaries are generally more precise than FFQs or 24 h recalls for a specific point-in-time analysis of patients’ quality of life rather than a generic FFQ that refers to usual intakes. Weekly food diaries also helped to reduce recall bias more typical of FFQs, which rely on good memory and knowledge of average intakes; therefore, they require abilities for quick mental calculations [79]. Moreover, greater precision in measurements is possible with food diaries because foods can be weighed on a kitchen scale.

Our analysis focuses mainly on diet, which may be a limitation as QoL is also influenced by physical activity, age, sleep quality, family support, financial difficulties, comorbidities and oncological treatments. However, our statistical model was adjusted for most of these variables.

## 5. Conclusions

In conclusion, we found an association between higher diet quality and better quality of life in women with breast cancer, contributing to the evidence that diet may impact overall quality of life and specific aspects such as fatigue and pain. In particular, our work shows how a higher diet quality, assessed by the HEI score, is associated with better health-related quality of life scores, both as individual dimensions as a summary index, as SumSc. Therefore, to confirm these results, more randomized controlled prospective studies are needed to evaluate the effect of a healthy dietary pattern on quality of life in an oncological context.

The long-term results of the main trial will be able to confirm or disprove these findings. In future studies, a lifestyle multidisciplinary approach may be advised as it has been shown to improve post-surgery breast cancer patients’ quality of life [80].

## Figures and Tables

**Table 1:** Average values of the total HEI score and its main components among participants (*n* = 492).

Components	Max Score	Mean Score (SD)
Adequacy ^a^		
Total Vegetables	5	3.2 (1.5)
Greens and Beans	5	4.2 (1.5)
Total Fruits	5	4.0 (1.5)
Whole Fruits	5	4.3 (1.4)
Whole Grains	10	3.0 (3.1)
Dairy	10	4.5 (3.3)
Total Protein Foods	5	4.6 (0.8)
Seafood and Plant Protein	5	4.4 (1.2)
Fatty Acids ^b^	10	3.8 (3.6)
Moderation ^c^		
Refined Grains	10	6.4 (3.0)
Sodium	10	9.4 (1.5)
Added Sugars	10	9.9 (0.2)
Saturated Fats	10	7.2 (2.6)
Total HEI-2015 score	100	68.8 (11.2)

HEI-2015 method details can be found in “Overview & Background of The Healthy Eating Index” (https://epi.grants.cancer.gov/hei/, accessed on 1 April 2022). ^a^ Adequacy components: dietary components involving food to eat more of for overall good health; ^b^ Fatty Acids Ratio: (Total MUFA + Total PUFA)/Total SFA. ^c^ Moderation components: components to limit or consume in small amounts for good health.

**Table 2:** Characteristics of the study participants by HEI-2015 median values.

Categories	Healthy Eating Index-2015 Score		*p*-Value
	<68.7 (*n* = 246)	≥68.7 (*n* = 246)	
Age (years)			0.304
≤50 yrs	69 (28%)	59 (24%)	
>50 yrs	177 (72%)	187 (76%)	
BMI (kg/m^2^)			**<0.001**
<25.0 *	74 (30%)	115 (47%)	
25.0 < 29.9	87 (35%)	62 (25%)	
30 < 34.9	52 (21%)	40 (16%)	
35 < 39.9	26 (11%)	19 (8%)	
≥40.0	10 (4%)	7 (3%)	
Smoking status			**<0.001**
No smoker	96 (39%)	135 (55%)	
Former smoker	83 (34%)	77 (31%)	
Smoker	67 (27%)	34 (14%)	
Civil status ^a,b^			0.453
Single	45 (18%)	51 (21%)	
In common law	201 (82%)	192 (79%)	
Education level (years) ^a^			**0.014**
≤11 yrs	103 (42%)	76 (31%)	
>11 yrs	143 (58%)	168 (69%)	
Step Count (steps/day)			0.104
<2500	52 (21%)	38 (15%)	
2500–4999	132 (54%)	128 (52%)	
≥5000	62 (25%)	80 (33%)	
Menopausal status			0.352
Menopausal	222 (90%)	222 (90%)	
Perimenopausal	9 (4%)	14 (6%)	
Premenopausal	15 (6%)	10 (4%)	
Cardiometabolic risk factors			0.882
None	145 (59%)	146 (59%)	
One	66 (27%)	62 (25%)	
More	35 (14%)	38 (15%)	
Cancer stage			0.882
I	72 (29%)	68 (28%)	
II	142 (58%)	143 (58%)	
III	32 (13%)	35 (14%)	
Molecular subtypes			0.356
Luminal A	74 (30%)	87 (35%)	
Luminal B	125 (51%)	116 (47%)	
HER2+	13 (5%)	7 (3%)	
TN	34 (14%)	36 (15%)	
Time from surgery (months)			0.652
<8 months	131 (53%)	126 (51%)	
≥8 months	115 (47%)	120 (49%)	
Surgery type ^a^			0.205
Quadrantectomy	192 (78%)	180 (73%)	
Mastectomy	53 (22%)	65 (27%)	
Neo-adjuvant chemotherapy			0.880
Never	221 (90%)	222 (90%)	
Completed	25 (10%)	24 (9.8%)	
Adjuvant chemotherapy			0.485
Ongoing	48 (20%)	39 (16%)	
Completed (<2 months)	36 (15%)	32 (13%)	
Completed (≥2 months)	80 (33%)	78 (32%)	
Never	82 (33%)	97 (39%)	
Radiotherapy ^a^			0.707
Ongoing	24 (10.0%)	19 (7.8%)	
Never	95 (39%)	97 (40%)	
Completed	122 (51%)	127 (52%)	

^a^ The sum does not add up to the total because of missing values, ^b^ Singles included widows, unmarried and divorced, * 3 underweight (BMI < 18.5) are included, BMI categories are: normal weight (<25.0), overweight (25.0 < 29.9), obesity grade I (30 < 34.9), obesity grade II (35 < 39.9) and obesity grade III (≥40.0). Wilcoxon rank sum test, *p*-value significance level <0.05.

**Table 3:** EORTC QLQ C30 dimensions mean scores and their association with HEI-2015.

	EORTC QLQ C30 Scores		Multivariable Analysis ^b^		
	Dimension	Mean (SD) ^a^	β ^c^	95% C.I.	*p*-Value
Functional Scales	Physical	82.6 (15.5)	0.06	−0.01; 0.13	0.089
	Role	80.0 (22.5)	0.01	−0.01; 0.13	0.734
	Emotional	74.7 (21.2)	0.05	0.00; 0.09	0.065
	Cognitive	82.5 (20.3)	0.02	−0.03; 0.07	0.387
	Social	77.4 (25.4)	0.04	0.00; 0.08	0.054
Symptoms Scales	Fatigue	32.4 (22.6)	−0.06	−0.11; −0.02	**0.009**
	Pain	23.4 (22.1)	−0.05	−0.10; −0.01	**0.028**
	Nausea and vomiting	6.4 (12.8)	−0.03	−0.11; 0.05	0.503
	Dyspnea	17.2 (22.3)	−0.07	−0.12, −0.02	**0.006**
	Insomnia	29.3 (27.9)	−0.01	−0.05, 0.02	0.495
	Constipation	15.4 (24.7)	0.01	−0.04, 0.05	0.703
	Appetite loss	6.2 (15.7)	−0.02	−0.09, 0.05	0.536
	Diarrhea	7.8 (16.4)	−0.09	−0.15, −0.03	**0.005**
	Financial difficulties	17.8 (26.7)	−0.04	−0.08, −0.01	**0.024**
Quality of life	Global Health Status	64.0 (20.5)	0.03	−0.02, 0.09	0.195

^a^ Mean values out of a 100 maximum score and standard deviation; ^b^ Multivariable linear regression models (LM) with HEI-2015 scores; ^c^ β coefficients were adjusted for geographical area, age, education, civil status, smoking status, steps, cardiometabolic risk factors, menopausal status, surgery type, time since surgery, cancer stage, chemotherapy and radiotherapy status). Bold *p*-values refer to the 95% confidence level significance.

**Table 4:** Adjusted regression for SumSc and HEI-2015 components.

Variable	Median * (IQR)	Exp(β)	95%CI	*p*-Value
Total HEI-2015 Score	68.7 (62.0, 76.2)	1.04	1.01, 1.07	**0.018**
Total vegetables	3.2 (1.9, 4.7)	1.02	1.00, 1.05	0.086
Beans and greens	5.0 (4.1, 5.0)	1.04	1.01, 1.07	**0.015**
Total fruits	5.0 (3.2, 5.0)	1.01	0.98, 1.04	0.667
Whole fruits	5.0 (4.7, 5.0)	1.01	0.97, 1.04	0.383
Whole grains	2.0 (0.0, 4.9)	1.02	0.99, 1.05	0.296
Dairy products	3.9 (1.3, 7.3)	1.01	0.98, 1.04	0.598
Total proteins foods	5.0 (4.5, 5.0)	0.99	0.96, 1.02	0.538
Seafood and plant proteins	5.0 (4.6, 5.0)	1.02	0.98, 1.05	0.311
Fatty acids	2.0 (1.7, 2.5)	1.01	0.98, 1.04	0.540
Refined grains	6.6 (4.1, 9.6)	1.02	0.99, 1.04	0.293
Sodium	10.0 (10.0, 10.0)	1.01	0.97, 1.04	0.701
Added sugars	10.0 (10.0, 10.0)	0.99	0.88, 1.12	0.916
Saturated fats	7.7 (5.5, 9.7)	0.97	0.95, 1.00	0.063

* Variables were categorized by median, with below-median being the model’s reference. β coefficients were adjusted for geographical area, age, education, civil status, smoking status, steps, cardiometabolic risk factors, menopausal status, surgery type, time since surgery, cancer stage, chemotherapy and radiotherapy status). Bold *p*-values refer to the 95% confidence level significance.

**Table 5:** Independent contribution of selected variables to HEI-2015 score and SumSc in an adjusted multivariate regression model.

Characteristic	Exp(Beta)	95%CI	*p*-Value *
HEI-2015 Score			**0.033**
<68.7	—	—	
≥68.7	1.03	1.00, 1.06	
Age (years)	1.00	1.00, 1.00	0.322
Education	1.00	1.00, 1.00	0.939
Geographical area			0.449
Aviano	—	—	
Campania	0.99	0.95, 1.03	
Sicilia	0.97	0.91, 1.02	
Civil status			0.861
Single	—	—	
In common law	1.00	0.96, 1.03	
Menopausal status			0.210
Menopausal	—	—	
Peri-menopausal	1.00	0.93, 1.07	
Pre-menopausal	1.06	0.99, 1.13	
Smoking status			0.052
No smoker	—	—	
Former smoker	0.98	0.95, 1.01	
Smoker	1.03	0.99, 1.07	
Physical activity			**<0.001**
<2500 steps/day	—	—	
2055–4999	1.05	1.01, 1.09	
≥5000 steps/day	1.09	1.04, 1.14	
Cancer Stage			0.442
I	—	—	
II	0.98	0.95, 1.02	
III	0.97	0.93, 1.02	
Type of surgery			0.770
Mastectomy	—	—	
Quadrantectomy	0.99	0.96, 1.03	
Time from surgery			0.417
<8 mths	—	—	
≥8 mths	1.01	0.98, 1.05	
Cardiometabolic risk factors			0.092
None	—	—	
One	0.96	0.93, 1.00	
More	0.98	0.94, 1.03	
Chemotherapy			**0.010**
Ongoing	—	—	
<2 mths	1.01	0.96, 1.07	
≥2 mths	1.09	1.03, 1.15	
Never	1.06	1.01, 1.12	
Radiotherapy			0.055
Ongoing	—	—	
Finished	1.08	1.01, 1.14	
Never	1.05	0.99, 1.11	

* Referring to z-test: bold values indicate a statistical significance at 95% level.

## Data Availability

The datasets analyzed (https://doi.org/10.5281/zenodo.16736903, accessed on 18 April 2024) during the current study are available from the corresponding author on reasonable request.

## Supplementary Materials

The following supporting information can be downloaded at: https://www.mdpi.com/article/10.3390/cancers16142576/s1, Figure S1. Healthy Eating Index construction model (HEI-2015). Table S1. HEI-2015 food groups features. Table S2. Medians and interquartile ranges of QLQ-C30 dimensions. Figure S2. Distribution of the HEI-2015 scores and C30 Summary Scores.

## References

[1] Bray F., Laversanne M., Sung H., Ferlay J., Siegel R.L., Soerjomataram I., Jemal A. (2024). Global cancer statistics 2022: GLOBOCAN estimates of incidence and mortality worldwide for 36 cancers in 185 countries. CA A Cancer J. Clin..

[2] Garcia Martin A., Fernandez Rodriguez E.J., Sanchez Gomez C., Rihuete Galve M.I. (2022). Study on the Socio-Economic Impact of Cancer Disease on Cancer Patients and Their Relatives. Healthcare.

[3] Naughton M.J., Weaver K.E. (2014). Physical and mental health among cancer survivors: Considerations for long-term care and quality of life. North Carol. Med. J..

[4] Niedzwiedz C.L., Knifton L., Robb K.A., Katikireddi S.V., Smith D.J. (2019). Depression and anxiety among people living with and beyond cancer: A growing clinical and research priority. BMC Cancer.

[5] Sung H., Ferlay J., Siegel R.L., Laversanne M., Soerjomataram I., Jemal A., Bray F. (2021). Global Cancer Statistics 2020: GLOBOCAN Estimates of Incidence and Mortality Worldwide for 36 Cancers in 185 Countries. CA A Cancer J. Clin..

[6] Rock C.L., Thomson C.A., Sullivan K.R., Howe C.L., Kushi L.H., Caan B.J., Neuhouser M.L., Bandera E.V., Wang Y., Robien K. (2022). American Cancer Society nutrition and physical activity guideline for cancer survivors. CA A Cancer J. Clin..

[7] World Cancer Research Fund International, American Institute for Cancer Research (2018). Diet, Nutrition, Physical Activity and Cancer: A Global Perspective: A Summary of the Third Expert Report.

[8] Salander P., Lilliehorn S., Hamberg K., Kero A. (2011). The impact of breast cancer on living an everyday life 4.5–5 years post-diagnosis—A qualitative prospective study of 39 women. Acta Oncol..

[9] Vandendorpe B., Drouet Y., Ramiandrisoa F., Guilbert P., Costa B., Servagi-Vernat S. (2021). Psychological and physical impact in women treated for breast cancer: Need for multidisciplinary surveillance and care provision. Cancer Radiother..

[10] Carreira H., Williams R., Muller M., Harewood R., Stanway S., Bhaskaran K. (2018). Associations Between Breast Cancer Survivorship and Adverse Mental Health Outcomes: A Systematic Review. J. Natl. Cancer Inst..

[11] Fiszer C., Dolbeault S., Sultan S., Bredart A. (2014). Prevalence, intensity, and predictors of the supportive care needs of women diagnosed with breast cancer: A systematic review. Psycho-Oncol..

[12] Berger A.M., Gerber L.H., Mayer D.K. (2012). Cancer-related fatigue: Implications for breast cancer survivors. Cancer.

[13] Jensen M.P., Chang H.Y., Lai Y.H., Syrjala K.L., Fann J.R., Gralow J.R. (2010). Pain in long-term breast cancer survivors: Frequency, severity, and impact. Pain Med..

[14] Yi J.C., Syrjala K.L. (2017). Anxiety and Depression in Cancer Survivors. Med. Clin. N. Am..

[15] Wang X., Wang N., Zhong L., Wang S., Zheng Y., Yang B., Zhang J., Lin Y., Wang Z. (2020). Prognostic value of depression and anxiety on breast cancer recurrence and mortality: A systematic review and meta-analysis of 282,203 patients. Mol. Psychiatry.

[16] Izci F., Ilgun A.S., Findikli E., Ozmen V. (2016). Psychiatric Symptoms and Psychosocial Problems in Patients with Breast Cancer. J. Breast Health.

[17] Dijkshoorn A.B.C., van Stralen H.E., Sloots M., Schagen S.B., Visser-Meily J.M.A., Schepers V.P.M. (2021). Prevalence of cognitive impairment and change in patients with breast cancer: A systematic review of longitudinal studies. Psycho-Oncol..

[18] WHO WHO Quality of life: Measuring Quality of Life. https://www.who.int/tools/whoqol.

[19] Cella D., Nowinski C.J. (2002). Measuring quality of life in chronic illness: The functional assessment of chronic illness therapy measurement system. Arch. Phys. Med. Rehabil..

[20] Sonneborn-Papakostopoulos M., Dubois C., Mathies V., Hess M., Erickson N., Ernst T., Huebner J. (2021). Quality of life, symptoms and dietary habits in oncology outpatients with malnutrition: A cross-sectional study. Med. Oncol..

[21] Zainordin N.H., Talib R.A., Shahril M.R., Sulaiman S., Karim N.A. (2020). Dietary Changes and Its Impact on Quality of Life among Malay Breast and Gynaecological Cancer Survivors in Malaysia. Asian Pac. J. Cancer Prev..

[22] Porciello G., Montagnese C., Crispo A., Grimaldi M., Libra M., Vitale S., Palumbo E., Pica R., Calabrese I., Cubisino S. (2020). Mediterranean diet and quality of life in women treated for breast cancer: A baseline analysis of DEDiCa multicentre trial. PLoS ONE.

[23] Montagnese C., Porciello G., Vitale S., Palumbo E., Crispo A., Grimaldi M., Calabrese I., Pica R., Prete M., Falzone L. (2020). Quality of Life in Women Diagnosed with Breast Cancer after a 12-Month Treatment of Lifestyle Modifications. Nutrients.

[24] Fu M.R., Axelrod D., Guth A.A., Cleland C.M., Ryan C.E., Weaver K.R., Qiu J.M., Kleinman R., Scagliola J., Palamar J.J. (2015). Comorbidities and Quality of Life among Breast Cancer Survivors: A Prospective Study. J. Pers. Med..

[25] Lei Y.Y., Ho S.C., Kwok C., Cheng A., Cheung K.L., Lee R., Mo F.K.F., Yeo W. (2022). Association of high adherence to vegetables and fruits dietary pattern with quality of life among Chinese women with early-stage breast cancer. Qual. Life Res. Int. J. Qual. Life Asp. Treat. Care Rehabil..

[26] Rios T.C., de Oliveira L.P.M., da Costa M.L.V., da Silva Baqueiro Boulhosa R.S., Roriz A.K.C., Ramos L.B., Bueno A.A. (2021). A poorer nutritional status impacts quality of life in a sample population of elderly cancer patients. Health Qual. Life Outcomes.

[27] Clemente-Suarez V.J., Redondo-Florez L., Rubio-Zarapuz A., Martinez-Guardado I., Navarro-Jimenez E., Tornero-Aguilera J.F. (2022). Nutritional and Exercise Interventions in Cancer-Related Cachexia: An Extensive Narrative Review. Int. J. Environ. Res. Public. Health.

[28] Khorasanchi A., Nemani S., Pandey S., Del Fabbro E. (2022). Managing Nutrition Impact Symptoms in Cancer Cachexia: A Case Series and Mini Review. Front. Nutr..

[29] Gil A., Martinez de Victoria E., Olza J. (2015). Indicators for the evaluation of diet quality. Nutr. Hosp..

[30] Burggraf C., Teuber R., Brosig S., Meier T. (2018). Review of a priori dietary quality indices in relation to their construction criteria. Nutr. Rev..

[31] Krebs-Smith S.M., Pannucci T.E., Subar A.F., Kirkpatrick S.I., Lerman J.L., Tooze J.A., Wilson M.M., Reedy J. (2018). Update of the Healthy Eating Index: HEI-2015. J. Acad. Nutr. Diet..

[32] Orchard T.S., Andridge R.R., Yee L.D., Lustberg M.B. (2018). Diet Quality, Inflammation, and Quality of Life in Breast Cancer Survivors: A Cross-Sectional Analysis of Pilot Study Data. J. Acad. Nutr. Diet..

[33] Wayne S.J., Baumgartner K., Baumgartner R.N., Bernstein L., Bowen D.J., Ballard-Barbash R. (2006). Diet quality is directly associated with quality of life in breast cancer survivors. Breast Cancer Res. Treat..

[34] Zick S.M., Colacino J., Cornellier M., Khabir T., Surnow K., Djuric Z. (2017). Fatigue reduction diet in breast cancer survivors: A pilot randomized clinical trial. Breast Cancer Res. Treat..

[35] Gong X.H., Wang J.W., Li J., Chen X.F., Sun L., Yuan Z.P., Yu J.M. (2017). Physical exercise, vegetable and fruit intake and health-related quality of life in Chinese breast cancer survivors: A cross-sectional study. Qual. Life Res. Int. J. Qual. Life Asp. Treat. Care Rehabil..

[36] Dalwood P., Marshall S., Burrows T.L., McIntosh A., Collins C.E. (2020). Diet quality indices and their associations with health-related outcomes in children and adolescents: An updated systematic review. Nutr. J..

[37] Vajdi M., Farhangi M.A. (2020). A systematic review of the association between dietary patterns and health-related quality of life. Health Qual. Life Outcomes.

[38] Augustin L.S., Libra M., Crispo A., Grimaldi M., De Laurentiis M., Rinaldo M., D’Aiuto M., Catalano F., Banna G., Ferrau F. (2017). Low glycemic index diet, exercise and vitamin D to reduce breast cancer recurrence (DEDiCa): Design of a clinical trial. BMC Cancer.

[39] Adesokan H.K., Akanbi I.M., Akanbi I.O., Obaweda R.A. (2015). Pattern of antimicrobial usage in livestock animals in south-western Nigeria: The need for alternative plans: Original research. Onderstepoort J. Vet. Res..

[40] Banca Dati di Composizione degli Alimenti per Studi Epidemiologici in Italia (BDA). https://bda.ieo.it/.

[41] Dietary Guidelines for Americans. https://www.dietaryguidelines.gov/.

[42] Food Pattern Equivalent Database (FPED). https://www.ars.usda.gov/northeast-area/beltsville-md-bhnrc/beltsville-human-nutrition-research-center/food-surveys-research-group/docs/fped-overview/.

[43] National Center for Health Statistics What We Eat in America/National Health and Nutrition Examination Survey, 2017–2018. Healthy Eating Index-2015 Scores: U.S. Department of Agriculture, Center for Nutrition Policy and Promotion. https://www.fns.usda.gov/cnpp/hei-scores-americans.

[44] Aaronson N.K., Ahmedzai S., Bergman B., Bullinger M., Cull A., Duez N.J., Filiberti A., Flechtner H., Fleishman S.B., de Haes J.C. (1993). The European Organization for Research and Treatment of Cancer QLQ-C30: A quality-of-life instrument for use in international clinical trials in oncology. J. Natl. Cancer Inst..

[45] EORTC Quality of Life Scoring Manual. https://qol.eortc.org/manuals/.

[46] Husson O., de Rooij B.H., Kieffer J., Oerlemans S., Mols F., Aaronson N.K., van der Graaf W.T.A., van de Poll-Franse L.V. (2020). The EORTC QLQ-C30 Summary Score as Prognostic Factor for Survival of Patients with Cancer in the “Real-World”: Results from the Population-Based PROFILES Registry. Oncologist.

[47] Giesinger J.M., Kieffer J.M., Fayers P.M., Groenvold M., Petersen M.A., Scott N.W., Sprangers M.A., Velikova G., Aaronson N.K., Group E.Q.o.L. (2016). Replication and validation of higher order models demonstrated that a summary score for the EORTC QLQ-C30 is robust. J. Clin. Epidemiol..

[48] Epidemiology and Genomics Research Program Healthy Eating Index SAS Code. https://epi.grants.cancer.gov/hei/sas-code.html.

[49] Wirkner J., Weymar M., Low A., Hamm C., Struck A.M., Kirschbaum C., Hamm A.O. (2017). Cognitive functioning and emotion processing in breast cancer survivors and controls: An ERP pilot study. Psychophysiology.

[50] Campbell-Enns H., Woodgate R. (2015). The psychosocial experiences of women with breast cancer across the lifespan: A systematic review protocol. JBI Database Syst. Rev. Implement. Rep..

[51] Bjerkeset E., Rohrl K., Schou-Bredal I. (2020). Symptom cluster of pain, fatigue, and psychological distress in breast cancer survivors: Prevalence and characteristics. Breast Cancer Res. Treat..

[52] Montazeri A., Vahdaninia M., Harirchi I., Ebrahimi M., Khaleghi F., Jarvandi S. (2008). Quality of life in patients with breast cancer before and after diagnosis: An eighteen months follow-up study. BMC Cancer.

[53] Mokhtari-Hessari P., Montazeri A. (2020). Health-related quality of life in breast cancer patients: Review of reviews from 2008 to 2018. Health Qual. Life Outcomes.

[54] Pagano I.S., Gotay C.C. (2006). Modeling quality of life in cancer patients as a unidimensional construct. Hawaii Med. J..

[55] Lee E., Kady V., Han E., Montan K., Normuminova M., Rovito M.J. (2022). Healthy Eating and Mortality among Breast Cancer Survivors: A Systematic Review and Meta-Analysis of Cohort Studies. Int. J. Environ. Res. Public. Health.

[56] Trauchburg A., Schwingshackl L., Hoffmann G. (2023). Association between Dietary Indices and Dietary Patterns and Mortality and Cancer Recurrence among Cancer Survivors: An Updated Systematic Review and Meta-Analysis of Cohort Studies. Nutrients.

[57] Kim S., Haines P.S., Siega-Riz A.M., Popkin B.M. (2003). The Diet Quality Index-International (DQI-I) provides an effective tool for cross-national comparison of diet quality as illustrated by China and the United States. J. Nutr..

[58] Sohouli M.H., Buckland G., Clark C.C.T., Santos H.O., Athayde F.L., Sanati V., Janani L., Sajadian A.S., Zarrati M. (2023). The relationship between diet quality indices and odds of breast cancer in women: A case-control study. BMC Women’s Health.

[59] Bu Y., Qu J., Ji S., Zhou J., Xue M., Qu J., Sun H., Liu Y. (2022). Dietary patterns and breast cancer risk, prognosis, and quality of life: A systematic review. Front. Nutr..

[60] Pisegna J., Xu M., Spees C., Krok-Schoen J.L. (2021). Mental health-related quality of life is associated with diet quality among survivors of breast cancer. Support. Care Cancer.

[61] Lee E., Zhu J., Velazquez J., Bernardo R., Garcia J., Rovito M., Hines R.B. (2021). Evaluation of Diet Quality Among American Adult Cancer Survivors: Results From 2005-2016 National Health and Nutrition Examination Survey. J. Acad. Nutr. Diet..

[62] Kranz S., Hasan F., Kennedy E., Zoellner J., Guertin K.A., Shivappa N., Hebert J.R., Anderson R., Cohn W. (2022). Diet Quality and Dietary Inflammatory Index Score among Women’s Cancer Survivors. Int. J. Environ. Res. Public. Health.

[63] Norman K., Stobaus N., Pirlich M., Bosy-Westphal A. (2012). Bioelectrical phase angle and impedance vector analysis—Clinical relevance and applicability of impedance parameters. Clin. Nutr..

[64] Lu Shin K.N., Mun C.Y., Shariff Z.M. (2020). Nutrition Indicators, Physical Function, and Health-Related Quality of Life in Breast Cancer Patients. Asian Pac. J. Cancer Prev..

[65] Guenther P.M., Reedy J., Krebs-Smith S.M. (2008). Development of the Healthy Eating Index-2005. J. Am. Diet. Assoc..

[66] Shahril M.R., Sulaiman S., Shaharudin S.H., Akmal S.N. (2013). Healthy eating index and breast cancer risk among Malaysian women. Eur. J. Cancer Prev..

[67] Danko A., Naughton M., Spees C., Bittoni A.M., Krok-Schoen J.L. (2021). Diet Quality and the Number of Comorbidities Are Associated with General Health Among Older Female Cancer Survivors. J. Aging Health.

[68] Fard N.A., Morales G.D., Mejova Y., Schifanella R. (2021). On the interplay between educational attainment and nutrition: A spatially-aware perspective. EPJ Data Sci..

[69] Llaha F., Ribalta A., Arribas L., Bellver M., Roura E., Guillen-Rey N., Megias-Rangil I., Alegret-Basora C., Tresserra-Rimbau A., Zamora-Ros R. (2022). A Review of Web-Based Nutrition Information in Spanish for Cancer Patients and Survivors. Nutrients.

[70] Williams K., Beeken R.J., Fisher A., Wardle J. (2015). Health professionals’ provision of lifestyle advice in the oncology context in the United Kingdom. Eur. J. Cancer Care.

[71] Spiro A., Baldwin C., Patterson A., Thomas J., Andreyev H.J. (2006). The views and practice of oncologists towards nutritional support in patients receiving chemotherapy. Br. J. Cancer.

[72] Vitale S., Palumbo E., Polesel J., Hebert J.R., Shivappa N., Montagnese C., Porciello G., Calabrese I., Luongo A., Prete M. (2023). One-year nutrition counselling in the context of a Mediterranean diet reduced the dietary inflammatory index in women with breast cancer: A role for the dietary glycemic index. Food Funct..

[73] Caccialanza R., Cereda E., Pinto C., Cotogni P., Farina G., Gavazzi C., Gandini C., Nardi M., Zagonel V., Pedrazzoli P. (2016). Awareness and consideration of malnutrition among oncologists: Insights from an exploratory survey. Nutrition.

[74] Silva P. (2023). Food and Nutrition Literacy: Exploring the Divide between Research and Practice. Foods.

[75] Alkerwi A., Baydarlioglu B., Sauvageot N., Stranges S., Lemmens P., Shivappa N., Hebert J.R. (2017). Smoking status is inversely associated with overall diet quality: Findings from the ORISCAV-LUX study. Clin. Nutr..

[76] Tsuzaki J., Maskarinec G., Mapa V., Shvetsov Y.B., Park S.Y., Monroe K.R., Lim U., Le Marchand L., Boushey C.J. (2024). Diet Quality and Body Mass Index Over 20 Years in the Multiethnic Cohort. J. Acad. Nutr. Diet..

[77] Kord-Varkaneh H., Salehi-Sahlabadi A., Zarezade M., Rahmani J., Tan S.C., Hekmatdoost A., Rashidkhani B. (2020). Association between Healthy Eating Index-2015 and Breast Cancer Risk: A Case-Control Study. Asian Pac. J. Cancer Prev..

[78] Pano O., Martinez-Lapiscina E.H., Sayon-Orea C., Martinez-Gonzalez M.A., Martinez J.A., Sanchez-Villegas A. (2021). Healthy diet, depression and quality of life: A narrative review of biological mechanisms and primary prevention opportunities. World J. Psychiatry.

[79] Coates J., Colaiezzi B., Fiedler J.L., Wirth J., Lividini K., Rogers B. (2012). A program needs-driven approach to selecting dietary assessment methods for decision-making in food fortification programs. Food Nutr. Bull..

[80] Ruiz-Vozmediano J., Lohnchen S., Jurado L., Recio R., Rodriguez-Carrillo A., Lopez M., Mustieles V., Exposito M., Arroyo-Morales M., Fernandez M.F. (2020). Influence of a Multidisciplinary Program of Diet, Exercise, and Mindfulness on the Quality of Life of Stage IIA-IIB Breast Cancer Survivors. Integr. Cancer Ther..

