# Supplementary material for: Baseline Association between Healthy Eating Index-2015 and Health-Related Quality of Life in Breast Cancer Patients Enrolled in a Randomized Trial
Source: Cancers (Basel). 2024 Jul 18;16(14):2576. doi: 10.3390/cancers16142576 (PMC11274909; doi:10.3390/cancers16142576)

Supplementary Tables (ST)

ST1 - Healthy Eating Index construction model (HEI-2015)
ST2 - HEI-2015 food groups features
ST3 - Medians and interquartile ranges of QLQ-C30 dimensions
ST4 - Distribution of the HEI-2015 scores and C30 Summary Scores

ST1. Healthy Eating Index construction model (HEI-2015)

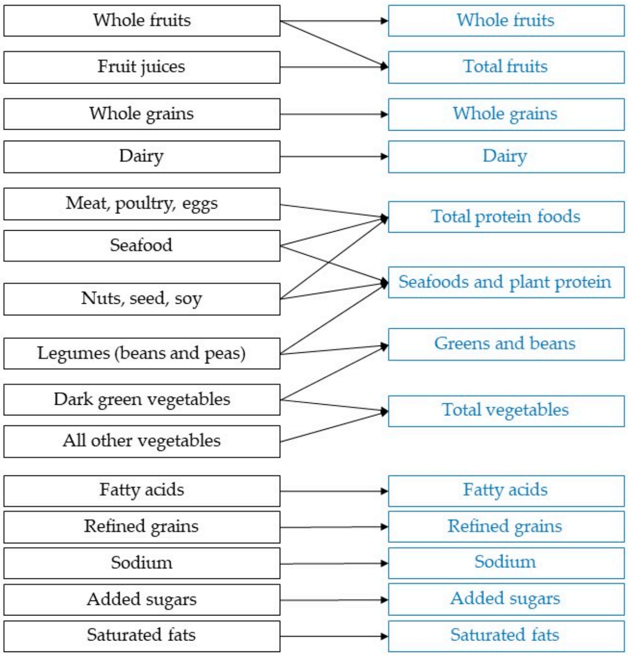

ST2 HEI-2015 food groups features

Total vegetables	*All form of vegetables (includes also tomatoes and carrots); brassicaceae ; potatoes; fresh peas*
Greens and beans	*Brassicaceae; legumes*
Whole fruits	*Fresh and dried fruits*
Total fruits	*Whole fruits; fruit juice; fruit jam*
Whole grains	*Bread, pasta and whole wheat biscuits*
Dairy	*All-milk products; yogurt, cheese, fortified soy beverages*
Total protein foods	*Meat (white, red, pork, processed, etc.); poultry; eggs; fish, shellfish, seafood; legumes; nuts, almonds, peanuts, seeds; soy (milk, products)*
Seafood and plant protein	*Fish, shellfish, seafood; legumes; nuts, almonds, peanuts, seeds; soy (milk, products)*
Refined grains	*All grains (not whole grains); pasta; egg pasta; biscuits; pizza*
Fatty acids	*Ratio of poly- and monounsaturated fatty acids to saturated fatty acids; (MUFA+PUFA)/SFA; in grams*
Sodium	*Sodium; in milligrams*
Added sugars	*All sugars added to foods (caloric sweeteners and syrups)*
Saturated fats	*Saturated fats total (SFA); in grams*

All food, group and nutrient data was obtained using WinFood software

ST3 Medians and interquartile ranges of QLQ-C30 dimensions

	QLQ C30 Dimension	Median (IQR)[a]
Functional Scales	Physical	86.7 (80.0, 93.3)
	Role	83.3 (66.7, 100.0)
	Emotional	75.0 (66.7, 91.7)
	Cognitive	83.3 (66.7, 100.0)
	Social	83.3 (66.7, 100.0)
Symptoms Scales	Fatigue	33.3 (22.2, 44.4)
	Pain	16.7 (0.0, 33.3)
	Nausea	0.0 (0.0, 16.7)
	Dyspnea	0.0 (0.0, 33.3)
	Insomnia	33.3 (0.0, 33.3)
	Constipation	0.0 (0.0, 33.3)
	Appetite loss	0.0 (0.0, 0.0)
	Diarrhoea	0.0 (0.0, 0.0)
	Financial	0.0 (0.0, 33.3)
Quality of life	Global Health Status	66.7 (50.0, 83.3)
	**QLQ C30 Summary Score**	
	C30 SumSc	84.3 (74.9, 90.8)

a Median and interquartile range; missing value are not shown;

ST4 Distribution of the HEI-2015 scores and C30 Summary Scores

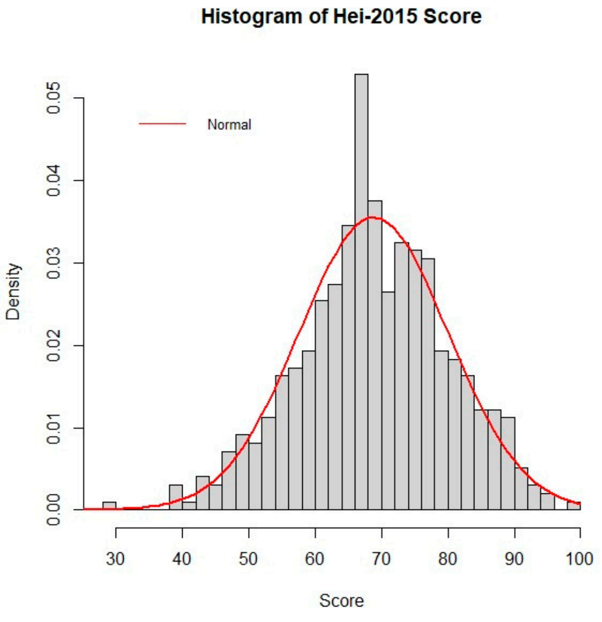

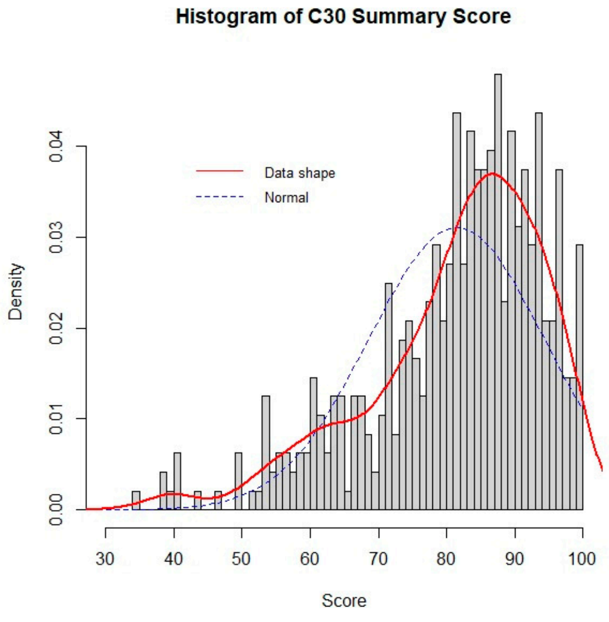

Supplement: Supplementary file 1 [file cancers-16-02576-s001.zip › cancers-3105363-supplementary.pdf]
